# Chemsex and Mental Health of Men Who Have Sex With Men in Germany

**DOI:** 10.3389/fpsyt.2020.542301

**Published:** 2020-11-04

**Authors:** Annette Bohn, Dirk Sander, Thorsten Köhler, Nico Hees, Felix Oswald, Norbert Scherbaum, Daniel Deimel, Henrike Schecke

**Affiliations:** ^1^Department of Psychology and Psychotherapy, Faculty of Health, University Witten-Herdecke, Witten, Germany; ^2^Department of Psychiatry and Psychotherapy, Medical Faculty, LVR-Hospital Essen, University of Duisburg-Essen, Essen, Germany; ^3^Department of Addictive Behavior and Addiction Medicine, Medical Faculty, LVR-Hospital Essen, University of Duisburg-Essen, Essen, Germany; ^4^Deutsche AIDS-Hilfe e.V., Berlin, Germany; ^5^German Institute for Addiction and Prevention Research, Catholic University of Applied Sciences, Köln, Germany; ^6^German Institute for Addiction and Prevention Research, Catholic University of Applied Sciences, Aachen, Germany

**Keywords:** chemsex, mental health, men who have sex with men, party and play (PNP), sexualised drug use, HIV

## Abstract

**Background:** Chemsex is defined as using certain substances immediately before or during sexual activities to facilitate, prolong and/or intensify sexual experience, mainly by some communities of men who have sex with men (MSM). Four substances are typically associated with chemsex: methamphetamine, mephedrone, GHB/GBL, and ketamine. While there is a lot of evidence for increased prevalence of HIV, sexually transmitted infections and other sexual health measures among MSM, who engage in chemsex, there has been little research on mental health aspects. This study aims to describe aspects of mental health among a sample of German men who have sex with men (MSM) who engage in chemsex and to describe potentially adverse consequences of chemsex behavior.

**Method:** This paper refers to a subset of participants from the *German Chemsex Survey*, an MSM-community recruited, self-completed online survey with a self-selected convenience sample. The survey comprised 420 different items considering recreational substance use, substance use in sexual settings, mental health, sexual transmitted infections, adverse consequences of chemsex behavior, and experiences of non-consensual sex acts. A group of participants who used methamphetamine, mephedrone, GHB/GBL, and/or ketamine in a sexual setting in the last 12 months (*n* = 280, chemsex group) was analyzed regarding symptoms of depression (PHQ-9), general anxiety disorder (GAD-7), somatization (PHQ-15), and PTSD (Primary Care PTSD Screen). Group comparisons were conducted between the chemsex group and men who did not use substances in a sexual context (*n* = 177, non-chemsex group). Mean scores of mental health measures were compared, as well as scores above a cut-off that indicates clinically relevant symptoms. Logistical regression was utilized to determine whether mental health measures can predict adverse consequences of engagement in chemsex behaviors.

**Results:** A total of 1,583 men started the survey; 1,050 participants provided information on substance use. Twenty-seven percent of participants (*n* = 280) reported that they used methamphetamine, mephedrone, GHB/GBL and/or ketamine in a sexual setting in the last 12 months. The chemsex group showed significantly higher mean scores for depression, anxiety, and somatization than the non-chemsex group, but effect sizes were low. Even though mean scores were heightened, they were still far below the cut-off for clinically relevant symptoms. The chemsex group reported significantly higher incidences of non-consensual sex acts compared with the non-chemsex group. Some men in the chemsex-group experienced potentially adverse consequences, such as loss of control regarding time and money spent for chemsex activities or amount of substances used at one occasion (49.6%), negative impacts on social functioning (33.6%), psychotic symptoms (13.2%), and physically aggressive behavior toward others (2.9%). Clinically relevant symptoms did not predict a higher likelihood for adverse consequences.

**Discussion:** Mean scores for depression, anxiety, and somatization were significantly higher in the chemsex-group, but effect sizes were low. Both groups reported poorer mental health compared to men in the German general population. Mental health measures did not contribute to predict potentially adverse consequences of chemsex behavior.

## Background

Chemsex is defined as using certain substances immediately before or during sexual activity to facilitate, prolong, and/or intensify sexual experience mainly by some communities of men who have sex with men (MSM) (1). There are four substances typically associated with chemsex: methamphetamine (“crystal meth,” “T,” “Tina”), mephedrone, GHB/GBL (“liquid ecstasy”), and ketamine (2, 3).

Previous studies have shown that MSM who engage in chemsex show a variety of distinctive features regarding their sexual behavior and their sexual health, including a higher likelihood to be HIV positive than MSM who do not engage in chemsex (4–6), as well as higher rates of sexually transmitted infections (STIs) (6, 7) and higher rates of hepatitis C infections (5, 7). Chemsex is also associated with engagement in group sex, having multiple sexual partners (8, 9) and more high-risk sexual behavior like condomless anal intercourse by HIV-negative men with partners that are HIV-positive or whose serostatus is unknown (10, 11).

In contrast, there has been considerably less research concerning the mental health status of MSM who engage in chemsex. Identifying as gay, bisexual or another non-heterosexual identity generally carries a higher risk for poor mental health compared to the general population, resulting in higher rates of depression and anxiety, suicide, and substance use disorders (12). This connection is often explained by the minority stress model (13). The model states that the connection between a non-heterosexual identity and higher incidences of mental health issues is mediated by ongoing stress and perceived and enacted stigma as a result of being part of a minority. Correlates of depression in a study of 1,340 HIV negative MSM in the UK, who were recruited from sexual health clinics, were a younger age, bisexual or other plurisexual orientation and a greater number of recreational drugs used (14). MSM with depressive symptoms may also be more likely to report high–risk sexual practices (15) and STI diagnoses (14).

Engagement with the LGBT community, found to be a contributing factor to overall well-being of LGBT people (16, 17), also seems to heighten the probability of drug use in general and especially in a sexual context for MSM. There is possibly a different social norm regarding substance use in some MSM communities (18). It has been reported by MSM engaged in chemsex that substance use is common in their friendship- or social group (19, 20).

A recent study of 3,017 gay or bisexual MSM in Australia found no significant relationship between drug use in sexual settings and clinically relevant symptoms of depression or anxiety (indicated by scores of 10 or above on the PHQ-9 and GAD-7, respectively) (21). However, a risk factor for poor mental health in this study was perceiving one's own substance use as problematic, or it being viewed as problematic by others. The authors concluded that there does not seem to be a direct or straightforward connection between substance use and mental health among MSM. In the same sample, no significant differences were found in rates of clinically relevant depression and anxiety symptoms between MSM who had recently injected drugs and those who had not (22).

In a survey of 1,649 MSM from the UK those who used drugs in sexual settings had lower overall life satisfaction than other participants, but no significant differences in body image satisfaction and psychological distress (10).

Since a high rate of HIV infections is often found among MSM who engage in chemsex, the implications of an HIV-infection also have to be considered when determining their mental health status. HIV positive people experience a higher risk of poor mental health outcomes, particularly depression (23, 24). It has been shown that HIV positive MSM who engage in chemsex face higher risks for self-reported anxiety or depression, sexual risk behavior and STI-diagnoses than HIV positive MSM who do not practice chemsex (7). This finding supports the suggestion of complex interconnections between HIV status, chemsex behaviors, and mental health.

To describe these complex interdependencies, Singer has proposed the model of syndemics (25, 26). This model allows an explanation for the observation of harmful impacts that somatic diseases, mental health and social conditions may have on each other, exceeding their singular effects. Syndemics “are most likely to emerge under conditions of health inequality caused by poverty, stigmatization stress or structural violence” [(25), p. 941]. There are consistent findings that a higher number of syndemic factors is associated with a higher risk for high risk sexual behavior and HIV transmission.

The aim of this study was to examine the mental health of German MSM practicing chemsex. Up to now, no other European sample of men who practice chemsex has been studied in this regard. In addition, the evaluation of mental health is more comprehensive than in previous studies, including for the first time somatization symptoms and trauma measures. Eventually, experiences of non-consensual sex as well as adverse outcomes of chemsex practice were investigated, which have not been widely covered before.

## Methods

### Design and Sampling

The “German Chemsex Survey” was a self-completed online survey (September until December 2018). It was targeted at MSM who use substances, particularly in a sexual setting, and was advertised accordingly. Participants were recruited via free-of-charge advertising on “PlanetRomeo” (the most popular German MSM-dating website/smartphone application), postings on LGBT-related websites and social media channels, as well as HIV/sexual health clinics. The sample was a self-selected convenience sample. To be included, participants had to be at least 18 years of age, identify as male, be attracted to and/or have had sex with men and have sufficient knowledge of German to be able to complete the survey. There was no financial compensation for participating. For this study, a subset of the collected data was analyzed. The aim was to describe and examine a group that practices chemsex, defined by the four substances most closely associated with chemsex. To determine whether certain characteristics are tied to engagement in chemsex, a non-chemsex group was identified for comparison.

### Ethical Considerations

All data in the study was collected anonymously. Participants could withdraw from the study at any time. They were supplied with a list of drug counseling and sexual health support services at the end of the survey should their participation have raised questions or concerns. The Ethics Committee of the Medical Department of the University of Duisburg-Essen granted its approval for the study (number UDE-18-8209-B0).

### Measures

The survey consisted of 420 items covering demographic characteristics, recreational substance use, substance use in sexual settings, mental health, sexual behavior, sexually transmitted infections, social support, experiences of discrimination and stigmatization, internalized homonegativity, the “big five” personality factors, harm reduction strategies, quality of life, and health care service utilization. Mental health was assessed using the German version of the Patient Health Questionnaire (PHQ-D) (27) with its three subscales for depressive symptoms (PHQ-9) (28), generalized anxiety symptoms (GAD-7) (29), and somatization symptoms (PHQ-15) (30). In addition, participants were asked to complete the four-item Primary Care PTSD Screener (31, 32), the Life Events Checklist for DSM-5 (33, 34) that covers potentially traumatizing life events, and the first question of the Suicide Behaviors Questionnaire-Revised (35, 36) which records lifetime suicidal thoughts and attempts. Based on a previous study (37), questions were added assessing non-consensual acts during sexual encounters, experiences of violence in connection with sex and non-consensual drug use when using substances in sexual settings. There was also a list of possible negative consequences of chemsex use, that those who reported chemsex could choose from.

### Statistical Analysis

Data analysis was conducted using IBM SPSS Statistics 25.0. *P*-values of <0.05 were taken to indicate statistical significance. The analyses presented here compare two groups: (i) men who used at least one “chemsex substance” (methamphetamine, GHB/GBL, ketamine, or mephedrone) in a sexual setting in the previous 12 months (*n* = 280); and (ii) men who did not report any substance use in sexual settings (apart from alcohol and/or nicotine) in the previous 12 months (*n* = 177).

For group comparisons regarding numerical variables Mann-Whitney-*U* tests were used, since all tested attributes were not normally distributed. For significant results, effect size was calculated by Cohen's d. Chi-square tests were used to compare the distribution of categorical variables. For significant results, effect size was calculated by the Phi coefficient. To assess associations between psychopathology and adverse consequences after practicing chemsex, logistic regression models were conducted.

### Sample Characteristics

**Figure 1:**
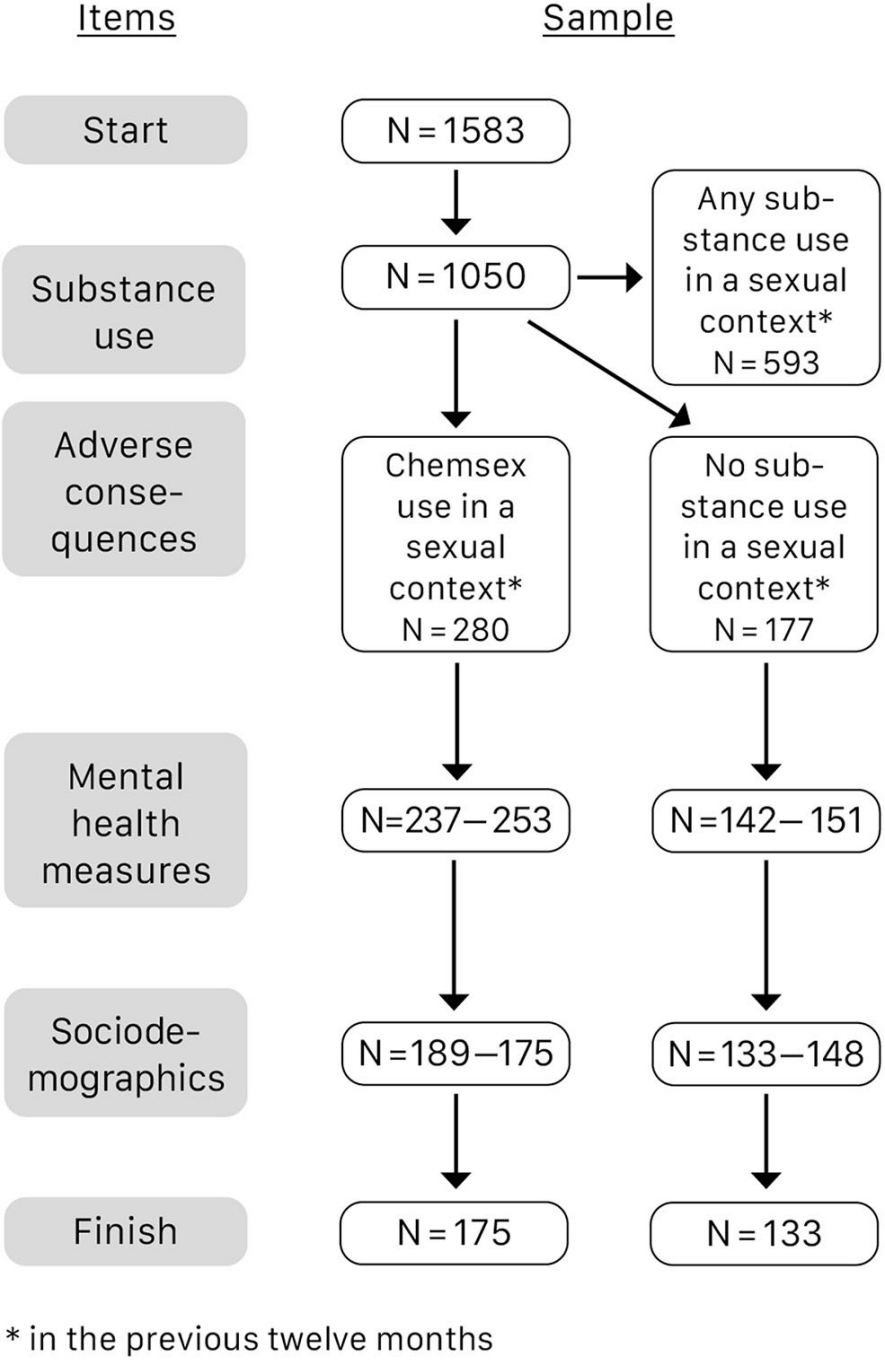
Development of participants' numbers in the course of the survey.

In total 1,583 people commenced the survey, 712 of whom completed all questions (45.0%). Data of non-completers were included on a pairwise basis, resulting in a different number of responses per analysis. Two hundred and eighty participants met the criterion of having used at least one of the four chemsex substances in a sexual context within the previous 12 months. One-hundred and five of the 280 men who reported chemsex (37.5%) dropped out at various points. Forty-four people from the 177 men who did not use any substances apart from alcohol and/or nicotine in a sexual setting also did not completely finish the survey (24.9%). See Figure 1 for a flowchart of development of participants' numbers in the course of the survey.

For details on the sample demographics for the chemsex and the non-chemsex group, see Table 1. Group comparisons were conducted to determine whether both groups are comparable regarding their demographics. A *t-*test showed that people in the non-chemsex group were significantly younger. Chi-square tests showed significant differences between the groups regarding country of birth, employment status and monthly net income. For employment status, a *post-hoc* test showed significantly more university students in the non-chemsex group. A *post-hoc* test for monthly net income showed that in the non-chemsex group there were significantly more people that earned < 1.000 Euros per month. All other attributes were evenly distributed.

**Table 1:** Sample demographics.

**Variable**	**Chemsex group**		**Non-chemsex group**		***p*-value**
	***N***	**M (SD)**	***N***	**M (SD)**	***t*-test**
Age	189	40.22 (10.66)	146	37.60 (12.62)	0.045^*^
	***N***	**%**	***N***	**%**	***χ***^**2**^
Gender identity	280		173		0.153
Male	277	98.9	168	97.1	
Transgender man	3	1.1	5	2.9	
Sexual identity	277		168		0.325
Gay/Homosexual	256	92.4	151	89.9	
Bisexual	15	5.4	15	8.9	
Queer	6	2.2	2	1.2	
Relationship status	263		161		
Single	112	42.6	77	47.8	0.292
In a relationship	151	57.4	84	52.2	
Country of birth	188		145		0.024^*^
Germany	155	82.4	132	91.0	
Other^a^	33	17.6	13	9.0	
Employment status	189		147		0.022^*^
Full-time employed	127	67.2	92	62.2	
Part-time employed	20	10.6	12	8.3	
Retired	11	5.8	5	3.5	
Student^b^	10	5.3	25	17.0	
Unemployed	9	4.8	6	4.2	
Other	12	6.3	7	4.8	
Monthly net income	188		144		0.022^*^
<1.000 Euros^b^	24	12.8	36	25.0	
1.000–2.000 Euros	63	33.5	49	34.0	
2.000–3.000 Euros	50	26.6	33	22.9	
More than 3.000 Euros	51	27.1	26	18.1	
Highest school leaving certificate	175		133		0.431
University or university of	136	77.7	102	76.7	
applied sciences entrance diploma					
General certificate of	27	15.4	19	14.3	
secondary education					
Certificate of secondary education	12	6.9	10	7.5	
Other/none	0	0.0	2	1.5	
Substance use 12 months in	280				
a sexual context					
Amyl nitrite (Poppers)	246	87.9			
Medication for erectile dysfunction	213	76.1			
GHB/GBL	206	73.6			
Alcohol	202	72.1			
Ecstasy	167	59.6			
Amphetamines	161	57.5			
Ketamine	156	55.7			
Methamphetamine	130	46.4			
THC	149	53.2			
Cocaine	122	43.6			
Mephedrone	98	35.0			
Opioid analgesics	15	5.4			
Heroin	3	1.1			
Substances injected in a sexual	84				
context 12 months					
Methamphetamine	72	85.7			
Mephedrone	34	40.5			
Ketamine	30	35.7			
Substance use 12 months not in	280		173		
a sexual context					
Alcohol	266	95.0	135	78.3	
Amyl nitrite (Poppers)	250	89.3	0	0.0	
Medication for erectile dysfunction	216	77.1	0	0.0	
GHB/GBL	209	74.6	0	0.0	
Ecstasy	193	68.9	2	1.2	
THC	186	66.4	20	11.8	
Amphetamines	186	66.4	0	0.0	
Ketamine	175	62.5	1	0.6	
Cocaine	141	50.3	0	0.0	
Methamphetamine	134	47.9	0	0.0	
Mephedrone	107	38.2	0	0.0	
Opioid analgesics	32	11.4	7	4.1	
Heroin	5	17.9	0	0.0	

* *p < 0.05.*

a *There were no clusters of non-German countries of birth.*

b *Attribute that differed significantly according to post-hoc test*.

Eighty four participants from the chemsex group (30.0%) reported having injected at least one of the substances. For substance used in a sexual setting by the whole chemsex sample and by IV substance users see Table 1.

## Results

For an overview of all group comparisons, see Table 2.

**Table 2:** Group comparisons between chemsex group and non-chemsex group, Mann-Whitney-*U* test for metric variable, Chi-square test for categorical variables.

**Variable**	**Chemsex group**		**Non-chemsex group**		**Test statistic**	**Significance**	**Effect size**
	***N***	**Mdn (IQR)**	***N***	**Mdn (IQR)**	**Mann-Whitney-U**	***p*-value**	***r***
PHQ-9 score	253	4.00 (4.00)	150	3.00 (4.25)	14725.5	0.000^*^	0.18
GAD-7 score	242	3.00 (4.00)	149	2.00 (3.00)	15648.0	0.029^*^	0.11
PHQ-15 score	252	5.00 (5.00)	151	3.00 (4.00)	15807.0	0.004^*^	0.14
Number of traumatic events	237	2.00 (2.00)	142	1.00 (2.00)	14749.5	0.023^*^	0.11
	***N***	**%**	***N***	**%**	**χ**^**2**^	***p*****-value**	**Phi**
PHQ-9 score ≥ 10	253	11.9	150	12.0	0.002	0.966	
GAD-7 score ≥ 10	242	8.3	149	8.7	0.025	0.874	
PHQ-15 score ≥ 10	252	13.5	151	10.6	0.729	0.393	
PTSD Screener score ≥ 3	234	11.5	139	12.9	0.164	0.686	
Suicide plans lifetime	251	12.7	152	14.5	0.243	0.622	
Suicide attempts lifetime	251	9.6	152	5.3	2.393	0.122	
Non-consensual sex acts	233	47.2	133	26.8	15.075	0.000^*^	0.194
Violence in a sexual setting	233	15.5	138	9.4	2.749	0.097	
HIV positive	199	41.2	96	13.5	22.700	0.000^*^	0.277
HIV status unknown	199	3.5	96	9.4	4.331	0.037^*^	0.121
Hepatitis C positive	198	2.0	70	0.0	a	0.576	
Hepatitis C status unknown	198	8.1	70	11.4	0.711	0.399	

a *Fisher's Exact Test was executed.*

* *p < 0.05*.

### Depressive Symptoms

The PHQ-9 scale has a maximum score of 27, with higher scores indicating higher levels of depressive symptoms. The chemsex sample's (*n* = 253) mean score on the PHQ-9 scale was 5.02 (*SD* = 4.14). 11.9% of the participants had a score of 10 or above and thus can be considered having clinically relevant depressive symptoms. PHQ-9 mean scores differed significantly in comparison to the non-chemsex group, with higher scores for the chemsex group. The groups did not differ regarding the distribution of clinically relevant symptoms.

### General Anxiety Symptoms

The GAD-7 scale has a maximum score of 21, with higher scores indicating higher levels of anxiety symptoms. The chemsex group participants' (*n* = 242) mean score on the GAD-7 scale was 3.81 (*SD* = 3.79), and 8.3 % of the sample had a score of 10 or above, suggesting clinically relevant anxiety symptoms. GAD-7 mean scores were significantly higher in the chemsex group, the distribution of clinically relevant symptoms did not differ between the groups.

### Somatization Symptoms

The PHQ-15 scale has a maximum score of 30, with higher scores indicating higher levels of somatization symptoms. The chemsex sample's (*n* = 252) PHQ-15 mean score was 5.14 (*SD* = 3.79), and 13.5% of the sample showed clinically relevant symptoms as indicated by a score of 10 or higher. There were significantly higher PHQ-15 mean scores in the chemsex group than in the non-chemsex group. The groups did not differ regarding the distribution of clinically relevant symptoms.

### Trauma

76.8% of all the chemsex group (*n* = 237) reported experiencing at least one potentially traumatizing event from a list of twelve. Most commonly, these were a serious accident (36.9%), a life-threatening illness (36.4%), physical violence from an unknown person (36.9%) or physical violence from a known person (34.3%). The mean number of events experienced by the chemsex group was 1.87 (*SD* = 1.70), and 11.6% show clinically relevant symptoms of PTSD, as indicated by a score of 3 or above in the PTSD primary care screener. Participants from the chemsex group reported having experienced a traumatic event significantly more often than those from the non-chemsex group. The groups did not differ regarding the distribution of clinically relevant symptoms of PTSD.

### Suicidality

12.7% of participants from the chemsex group (*n* = 251) reported having planned for suicide at least once in their lifetime, 9.6% had actively attempted suicide at least once. The groups did not differ regarding suicide plans or attempts.

### Non-consensual Acts During Sex

In the chemsex group (*n* = 233), 47.2% reported having experienced their sexual partners not respecting their boundaries, which differs significantly from the non-chemsex group (*n* = 133; 26.8%). 15.5% of men engaging in chemsex (*n* = 233) reported the experience of violence in a sexual setting, which is not significantly different from the non-chemsex group (*n* = 138; 9.4%). 17.7% of the chemsex group (*n* = 234) reported that sexual partners had administered drugs to them without their consent.

### Infectious Diseases

41.2% of the chemsex group (*n* = 199) reported being HIV positive, 2.0% reported being infected with hepatitis C. Significantly more men from the chemsex group were HIV-positive than those from the non-chemsex group. There were no differences for the rates of unknown current HIV-status between the groups, as for the rates of hepatitis C-infections.

### Adverse Consequences of Use and Their Associations With Mental Health Measures

49.6% of participants from the chemsex group (*n* = 280) reported a loss control during or after a chemsex session in the last 12 months, meaning that they either spent more time or money on chemsex than they originally intended or that they could not entirely remember the event. 33.6% stated that they have been missing work or other appointments after a chemsex session or that they were still under the influence of drugs when working. 13.2% reported hearing voices or having paranoid experiences after engaging in chemsex. 2.9% have assaulted another person as an after-effect of a chemsex session.

Logistical regression analyses were conducted to determine whether those who show clinically relevant symptoms of depression, anxiety, somatization or PTSD are more likely to show any of the adverse consequences. For each model, the adverse consequence was taken as the outcome variable with the clinically developed symptoms as dichotomous predictors. None of the models showed a good fit, with Nagelkerke's *R*^2^ values of 0.160 (assault of another person), 0.092 (missing work or other appointments), 0.078 (hearing voices or having paranoid experiences), and 0.056 (spending more time or money or loss of memory). In all models, only two predictors turned out to be significant: clinically developed somatization symptoms predicted assaults (*p* = 0.033; *OR* = 5.653, CI:1.152–27.730) and anxiety symptoms predicted missing work or other appointments or going to work while still under the influence of drugs (*p* = 0.011; *OR* = 9.070, CI:1.667–49.334).

## Discussion

With regard to mental health measures, a direct comparison of the chemsex and non-chemsex group, found significant differences for the mean scores of depression, somatization, and anxiety, as well as lifetime number of traumatic events experienced, which were all higher for the chemsex group. No differences between the groups for the rates of clinically developed symptoms were found. Those who practice chemsex reported significantly more incidences of violation of their sexual boundaries as well as a higher rate of HIV infections, compared to those who do not practice chemsex.

### Mental Health

All mean scores of mental health measures were significantly higher for the chemsex group, but these differences show only small effect sizes, pointing to a weak interrelation. Comparing the distribution of clinically relevant symptoms between the different groups, there were no significant differences.

The prevalence rate of 11.9% for clinically relevant symptoms of depression in the chemsex group is almost twice that of the general male population in Germany (6.1%) (38), but comparable to a recent sample of MSM from the UK, which showed a rate of 12.4% with a PHQ-9 score ≥ 10 (14). In contrast to a study of Australian MSM that identified 28.3% with a PHQ-9 score ≥ 10 (21), this study's chemsex sample expresses fewer depressive symptoms over all. Compared to clinically relevant symptoms of somatization in the general population concerning 8.1% of people (30), the chemsex sample's rate of 13.5% was slightly higher. In comparison to the German general population's rate of 5.9% with clinically relevant symptoms of Generalized Anxiety Disorder (39), the chemsex sample's rate was only slightly higher, at 8.3%. Compared to 17.9% in the sample of 3,017 Australian MSM (21), the chemsex group seems to show comparably little symptoms of anxiety. The rate of 11.5% in the chemsex sample that screened positive for PTSD is considerably higher compared to the general population in Germany, for which a 12 months prevalence for PTSD in men of 0.9% was measured (40).

The number of lifetime suicide attempts reported by those engaged in chemsex was 9.6%. A recent study from Sweden found a percentage of lifetime suicide attempts for gay men of 10.0%, whereas merely 2.2% heterosexual men attempted suicide in their lifetime (41). In conclusion, the chemsex sample has a history of lifetime suicide attempts that is comparable to other MSM, but higher than in the general population.

We can observe some strain on those who practice chemsex compared to those who do not, as suggested by heightened mean scores for depression, somatization and trauma events. However, these differences are not reflected in the rates of clinically relevant symptoms. Overall, it seems that the chemsex group does not differ much from other MSM groups that aren't solely comprised of men who engage in chemsex. Previous research has suggested a complex interplay between substance use, sexual behavior and mental health measures, with various factors impacting on and influencing each other. There is no information about the sample's rate on substance dependency, which has a negative impact on mental health (21). The rate of 41.2% HIV positive chemsex participants also allows us to conclude that mental health may be negatively impacted (23, 24). These are all potential negative influences on mental health statuses.

It has also been shown that those who practice chemsex have closer ties to the LGBT community than other MSM (6, 19, 42), which has a positive effect on well-being (16, 17).

### Adverse Outcomes

About half of the participants that practice chemsex have experienced a loss of control during or after a chemsex session in the last 12 months, meaning that they either spent more time or money on chemsex than they originally intended or that they could not entirely remember the event. This adverse outcome could not be predicted by clinically developed symptoms of depression, anxiety, somatization, or PTSD. About a third stated that they have been missing work or other appointments after a chemsex session or that they were still under the influence of drugs when working. Clinically developed symptoms of anxiety showed to be a significant predictor for this outcome. About one in ten men reported hearing voices or having paranoid experiences after engaging in chemsex. This outcome could not be predicted by any clinically developed symptoms. Three percent of participants have assaulted another person as an after-effect of a chemsex session. This outcome could be predicted by clinically developed symptoms of somatization.

Overall, all models predicting negative outcomes showed low models-of-fit. Even though two significant predictors could be identified, the results have to be interpreted cautiously, since the rates of clinically relevant symptoms for all measures were lowly pronounced in this particular group, so the distribution is uneven. The rates of the adverse outcomes of assaulting someone and having paranoid experiences or hearing voices were also quite unevenly distributed, which further limits the models' explanatory power.

### Non-consensual Sex Acts

46.6% of chemsex users report non-consensual acts during sex, and violence during sex was experienced by 16.8%. Significantly more experiences of non-consensual acts were reported in the chemsex group compared to the non-chemsex group, of which 28.0% reported such incidents. A small effect size shows for this result. There were no significant differences regarding the experience of violence during sex for the different groups. A recent study from the UK that chose a similar sampling approach as this study, recruited MSM participants via Grindr, a dating app that is often used by MSM to find sex partners (43). Of these men, 37.7% reported having at least experienced one form of intimate partner violence. Being a victim of sexual intimate partner violence was significantly correlated with an increase of substance use in the last month, showing yet another interconnection relevant to the complex behavior of chemsex practice. There is more evidence that not only exposure to intimate partner violence as a victim, but also as a perpetrator, is associated with increased chance of substance use (44). Intimate partner violence is known as a syndemic risk factor (45).

According to a recent study the experience of non-consensual sex is far more common for MSM, with a rate of 22.8%, compared to 4.3% of men that have sex exclusively with women (46).

The study by Bourne et al. from 2014 regarding chemsex in the UK found anecdotal evidence of non-consensual sex associated with substance use, particularly in cases that men had accidentally overdosed. Interviewees in this study reported that there “was a particularly blurry line regarding consent in the context of chemsex” [(1), p. 59]. 17.7% of those practicing chemsex in this study also reported that sexual partners administered drugs to them without their consent. So far, there has been no systematic research regarding chemsex and consent, but these findings suggest the necessity of addressing the topic.

### HIV and Hepatitis C Infections

The rate of 41.2% HIV positive in the chemsex group is higher than the rate of HIV-positive German MSM in 2010, which was 8.0% (47). The HIV rate in the general population in Germany in 2015 was 0.1% (48). The distribution of HIV positive people in the chemsex group in this study differed significantly from the non-chemsex group with a medium effect size.

Those who injected chemsex substances showed a significantly higher incidence of HIV-infection, which is consistent with other studies' findings (22). Frequency of use was also relevant in this sample, with more frequent users showing higher rates of HIV. Practicing chemsex more often and injecting the drugs appear to be risk factors for HIV infection, and although this cannot be determined by this study's correlational data, there are findings pointing in this direction (5). Based on this, the use of pre exposure prophylaxis (PrEP) could be a useful strategy for those engaged in chemsex.

The overall rate of hepatitis C infections in the chemsex group was 2.0 %, which does not differ significantly from the non-chemsex group. The hepatitis C rate among the German general population is 0.3% (49).

### Limitations and Future Research

The limitations of this study include the cross-sectional design, which does not allow causal explanations, as well as the self-selected sample with high levels of income and education overall. It is possible that chemsex users do tend to have a certain socio-economic status but determining this would require a representative survey. The questionnaire was only available in German, excluding participants with insufficient German language skills. It was also promoted by Aidshilfe (the largest HIV/AIDS peer-support organization in Germany), so the high HIV rate in the sample might be a sampling effect. The study was targeted at substance users and advertised, respectively, so drug use was high overall throughout the sample and the findings should not be used to estimate a prevalence of substance use in the German MSM population.

Even though not all users reported consuming in a sexual setting, the identified non-chemsex group that did not practice sexualized drug use may still not be comparable to other non-chemsex MSM samples. There have also been found some demographic differences between the groups, which could put some restrictions on the comparison between the chemsex group and the non-chemsex group. The non-chemsex group was found to be younger with a higher rate of university students and people with low income. There were also more people not born in Germany in the chemsex sample. Since there was a dropout rate of 37.5% in the chemsex group and 22.2% in the non-chemsex group and all demographic data was retrieved at the very end of the study, there might be a bias concerning the demographic data.

For future research, it would be useful to study a more diverse group in terms of socio-economic status. Additionally, more research would be necessary to determine complex interrelations between the different factors, in order to assess which are risk factors for poor mental health, and in which situations. Another topic that would be useful to explore and study further is the relation between chemsex and non-consensual sex.

### Outlook

Support and treatment options for MSM who practice chemsex and want to reduce or quit their substance use are sparse so far. In previous studies, men have reported a hesitancy to attend regular drug counseling services or programs out of fear not be understood (50). There is a distinct need for counseling tailored to chemsex users and their needs which is not generally focused on abstinence, and which also incorporates harm reduction strategies (2). There are promising findings for German MSM, who use methamphetamine that some harm reduction strategies are already applied and well-accepted (51). In programs aimed at abstinence, the established connection between sex and substance use makes sexual situations potential triggers for relapse, and thus needs to be specifically addressed (37). There are treatment approaches integrating professional support as well as MSM peer-support approaches (50), which seem especially important, given the evidence that substance use is considered a social norm in some parts of the community (18, 22). There is also a need for well-trained and informed staff in sexual health and outreach clinics (52).

## Data Availability Statement

The datasets generated for this study are available on request to the corresponding author.

## Ethics Statement

The studies involving human participants were reviewed and approved by Ethics Committee of the Medical Department of the University of Duisburg-Essen, approval nr. UDE-18-8209-B0. The patients/participants provided their written informed consent to participate in this study.

## Author Contributions

AB article writing, data analysis, and literature search. HS study conceptualization, data analysis, and article writing. FO survey programming and data analysis. DS study conceptualization. TK consulting data analysis. NH and NS editing article. DD study conceptualization and editing article. All authors contributed to the article and approved the submitted version.

## Conflict of Interest

NS received honoraria for several activities (advisory boards, lectures, manuscripts) by AbbVie, Hexal, Janssen-Cilag, MSD, Medice, Mundipharma, Reckitt-Benckiser/Indivior, and Sanofi-Aventis. During the last 3 years, he participated in clinical trials financed by the pharmaceutical industry. The remaining authors declare that the research was conducted in the absence of any commercial or financial relationships that could be construed as a potential conflict of interest.

## References

[1] Bourne A Reid D Hickson F Torres Rueda S Weatherburn P. The Chemsex Study: Drug Use in Sexual Settings Among Gay and Bisexual Men in Lambeth, Southwark and Lewisham. Technical Report. London: Sigma Research, London School of Hygiene & Tropical Medicine (2014).

[2] Bourne A Reid D Hickson F Torres-Rueda S Steinberg P Weatherburn P. “Chemsex” and harm reduction need among gay men in South London. Int J Drug Policy. (2015) 26:1171–6. 10.1016/j.drugpo.2015.07.013 26298332

[3] Edmundson C Heinsbroek E Glass R Hope V Mohammed H White M. Sexualised drug use in the United Kingdom (UK): A review of the literature. Int J Drug Policy. (2018) 55:131–48. 10.1016/j.drugpo.2018.02.002 29625796

[4] Frankis J Flowers P McDaid L Bourne A. Low levels of chemsex among men who have sex with men, but high levels of risk among men who engage in chemsex: analysis of a cross-sectional online survey across four countries. Sexual Health. (2018) 15:144–50. 10.1071/SH17159 29592829 PMC6778053

[5] Pakianathan M Whittaker W Lee MJ Avery J Green S Nathan B. Chemsex and new HIV diagnosis in gay, bisexual and other men who have sex with men attending sexual health clinics. HIV Med. (2018) 19:485–90. 10.1111/hiv.12629 29790254

[6] Rosińska M Gios L Nöstlinger C Vanden Berghe W Marcus U Schink S. Prevalence of drug use during sex amongst MSM in Europe: Results from a multi-site bio-behavioural survey. Int J Drug Policy. (2018) 55:231–41. 10.1016/j.drugpo.2018.01.002 29402683

[7] Pufall EL Kall M Shahmanesh M Nardone A Gilson R Delpech V. Sexualized drug use (‘chemsex’) and high-risk sexual behaviours in HIV-positive men who have sex with men. HIV Med. (2018) 19:261–70. 10.1111/hiv.12574 29368440 PMC5900961

[8] Glynn RW Byrne N O'Dea S Shanley A Codd M Keenan E. Chemsex, risk behaviours and sexually transmitted infections among men who have sex with men in Dublin, Ireland. Int J Drug Policy. (2018) 52:9–15. 10.1016/j.drugpo.2017.10.008 29223761

[9] Sewell J Miltz A Lampe FC Cambiano V Speakman A Phillips AN. Poly drug use, chemsex drug use, and associations with sexual risk behaviour in HIV-negative men who have sex with men attending sexual health clinics. Int J Drug Policy. (2017) 43:33–43. 10.1016/j.drugpo.2017.01.001 28189979

[10] Hibbert MP Brett CE Porcellato LA Hope VD. Psychosocial and sexual characteristics associated with sexualised drug use and chemsex among men who have sex with men (MSM) in the UK. Sex Transm Infect. (2019) 95:342–50. 10.1136/sextrans-2018-053933 30979782

[11] Melendez-Torres GJ Hickson F Reid D Weatherburn P Bonell C. Findings from within-subjects comparisons of drug use and sexual risk behaviour in men who have sex with men in England. Int J STD AIDS. (2017) 28:250–8. 10.1177/0956462416642125 27013616

[12] King M Semlyen J Tai SS Killaspy H Osborn D Popelyuk D. A systematic review of mental disorder, suicide, and deliberate self harm in lesbian, gay and bisexual people. BMC Psychiatry. (2008) 8:70. 10.1186/1471-244X-8-70 18706118 PMC2533652

[13] Meyer IH. Prejudice, social stress, and mental health in lesbian, gay, and bisexual populations: conceptual issues and research evidence. Psychol Bull. (2003) 129:674–97. 10.1037/0033-2909.129.5.674 12956539 PMC2072932

[14] Miltz AR Rodger AJ Sewell J Speakman A Phillips AN Sherr L. Clinically significant depressive symptoms and sexual behaviour among men who have sex with men. BJPsych open. (2017) 3:127–37. 10.1192/bjpo.bp.116.003574 28507772 PMC5421094

[15] Alvy LM McKirnan DJ Mansergh G Koblin B Colfax GN Flores SA. Depression is associated with sexual risk among men who have sex with men, but is mediated by cognitive escape and self-efficacy. AIDS Behav. (2011) 15:1171–9. 10.1007/s10461-010-9678-z 20217471

[16] Durso LE Meyer IH. Patterns and predictors of disclosure of sexual orientation to healthcare providers among lesbians, gay men, and bisexuals. Sex Res Soc Policy. (2013) 10:35–42. 10.1007/s13178-012-0105-2 23463442 PMC3582401

[17] Kertzner RM Meyer IH Frost DM Stirratt MJ. Social and psychological weil-being in lesbians, gay men, and bisexuals: the effects of race, gender, age, and sexual identity. Am J Orthopsychiatry. (2009) 79:500–10. 10.1037/a0016848 20099941 PMC2853758

[18] Lea T Hammoud M Bourne A Maher L Jin F Haire B. Attitudes and perceived social norms toward drug use among gay and bisexual men in Australia. Subst Use Misuse. (2019). 54:944–54. 10.1080/10826084.2018.1552302 30648480

[19] Pollard A Nadarzynski T Llewellyn C. Syndemics of stigma, minority-stress, maladaptive coping, risk environments and littoral spaces among men who have sex with men using chemsex. Cult Health Sexual. (2018) 20:411–27. 10.1080/13691058.2017.1350751 28741417

[20] Smith V Tasker F. Gay men's chemsex survival stories. Sex Health. (2018) 15:116–22. 10.1071/SH17122 29268074

[21] Prestage G Hammoud M Jin F Degenhardt L Bourne A Maher L. Mental health, drug use and sexual risk behavior among gay and bisexual men. Int J Drug Policy. (2018) 55:169–79. 10.1016/j.drugpo.2018.01.020 29429865

[22] Bui H Zablotska-Manos I Hammoud M Jin F Lea T Bourne A. Prevalence and correlates of recent injecting drug use among gay and bisexual men in Australia: results from the FLUX study. Int J Drug Policy. (2018) 55:222–30. 10.1016/j.drugpo.2018.01.018 29429864

[23] Ciesla JA Roberts JE. Meta-analysis of the relationship between HIV infection and risk for depressive disorders. Am J Psychiatry. (2001) 158:725–30. 10.1176/appi.ajp.158.5.725 11329393

[24] Nanni MG Caruso R Mitchell AJ Meggiolaro E Grassi L. Depression in HIV infected patients: a review. Curr Psychiatry Rep. (2015) 17:530. 10.1007/s11920-014-0530-4 25413636

[25] Singer M Bulled N Ostrach B Mendenhall E. Syndemics and the biosocial conception of health. Lancet. (2017) 389:941–50. 10.1016/S0140-6736(17)30003-X 28271845

[26] Singer M Clair S. Syndemics and public health: reconceptualizing disease in bio-social context. Med Anthropol Q. (2003) 17:423–41. 10.1525/maq.2003.17.4.423 14716917

[27] Gräfe K Zipfel S Herzog W Löwe B. Screening psychischer Störungen mit dem “Gesundheitsfragebogen für Patienten (PHQ-D).” *Diagnostica*. (2004) 50:171–81. 10.1026/0012-1924.50.4.17119742274

[28] Kroenke K Spitzer RL Williams JBW. The PHQ-9: validity of a brief depression severity measure. J Gen Intern Med. (2001) 16:606–13. 10.1046/j.1525-1497.2001.016009606.x 11556941 PMC1495268

[29] Spitzer RL Kroenke K Williams JBW Löwe B. A brief measure for assessing generalized anxiety disorder: The GAD-7. Arch Intern Med. (2006) 166:1092–7. 10.1001/archinte.166.10.1092 16717171

[30] Kocalevent RD Hinz A Brähler E. Standardization of a screening instrument (PHQ-15) for somatization syndromes in the general population. BMC Psychiatry. (2013) 13:91. 10.1186/1471-244X-13-91 23514436 PMC3606198

[31] Cameron RP Gusman D. The primary care PTSD screen (PC-PTSD): development and operating characteristics. Primary Care Psychiatry. (2003) 9:9–14. 10.1185/135525703125002360

[32] Schäfer I Schulze C. Deutsche Version des “Primary CarePosttraumatic Stress Disorder screening questionnaire.” Universität Hamburg (2010).

[33] Ehring T Knaevelsrud C Krüger A Schäfer I. Life Events Checklist für DSM-5 (LEC-5): Deutsche Version. German version. Events Checklist for DSM-5 (Hamburg: LEC-5) (2014).

[34] Weathers F Blake D Schnurr P Kaloupek D Marx B Keane T. The Life Events Checklist for DSM-5 (LEC-5). (2013) Available online at: www.ptsd.va.gov (accessed June 15, 2020).

[35] Glaesmer H Kapusta ND Teismann T Wagner B Hallensleben N Spangenberg L. Psychometrische eigenschaften der deutschen version des suicide behaviors questionnaire revised (SBQ-R). Psychother Psycho Med Psychol. (2018) 68:346–52. 10.1055/s-0043-118335 28958123

[36] Osman A Bagge CL Gutierrez PM Konick LC Kopper BA Barrios FX. The suicidal behaviors questionnaire-revised (SBQ-R): validation with clinical and nonclinical samples. Assessment. (2001) 8:443–54. 10.1177/107319110100800409 11785588

[37] Deimel D Stöver H Hößelbarth S Dichtl A Graf N Gebhardt V. Drug use and health behaviour among German men who have sex with men: results of a qualitative, multi-centre study. Harm Reduc J. (2016) 13:36. 10.1186/s12954-016-0125-y 27938393 PMC5148887

[38] Busch M Maske U Ryl L Schlack R Hapke U. Prevalence of depressive symptoms and diagnosed depression among adults in Germany: results of the German health interview and examination survey for adults (DEGS1). Bundesgesundheitsblatt Gesundheitsforschung Gesundheitsschutz. (2013) 56:733–9. 10.1007/s00103-013-1688-3 23703492

[39] Hinz A Klein AM Brähler E Glaesmer H Luck T Riedel-Heller SG. Psychometric evaluation of the generalized anxiety disorder screener GAD-7, based on a large German general population sample. J Affect Disord. (2017) 210:338–44. 10.1016/j.jad.2016.12.012 28088111

[40] Jacobi F Höfler M Strehle J Mack S Gerschler A Scholl L. Mental disorders in the general population. Study on the health of adults in Germany and the additional module mental health (DEGS1-MH). Nervenarzt. (2014) 85:77–87. 10.1007/s00115-013-3961-y 24441882

[41] Björkenstam C Kosidou K Björkenstam E Dalman C Andersson G Cochran S. Self-reported suicide ideation and attempts, and medical care for intentional self-harm in lesbians, gays and bisexuals in Sweden. J Epidemiol Community Health. (2016) 70:895–901. 10.1136/jech-2015-206884 26945095 PMC6884315

[42] Graf N Dichtl A Deimel D Sander D Stöver H. Chemsex among men who have sex with men in Germany: motives, consequences and the response of the support system. Sexual Health. (2018) 15:151–6. 10.1071/SH17142 29580377

[43] Duncan DT Goedel WC Stults CB Brady WJ Brooks FA Blakely JS. A study of intimate partner violence, substance abuse, and sexual risk behaviors among gay, bisexual, and other men who have sex with men in a sample of geosocial-networking smartphone application users. Am J Mens Health. (2018) 12:292–301. 10.1177/1557988316631964 26873342 PMC5818104

[44] Buller AM Devries KM Howard LM Bacchus LJ. Associations between intimate partner violence and health among men who have sex with men: a systematic review and meta-analysis. PLoS Med. (2014) 11:e1001609. 10.1371/journal.pmed.1001609 24594975 PMC3942318

[45] Stall R Mills TC Williamson J Hart T Greenwood G Paul J. Association of co-occurring psychosocial health problems and increased vulnerability to HIV/AIDS among urban men who have sex with men. Am J Public Health. (2003) 93:939–42. 10.2105/AJPH.93.6.939 12773359 PMC1447874

[46] Mercer CH Prah P Field N Tanton C Macdowall W Clifton S. The health and well-being of men who have sex with men (MSM) in Britain: evidence from the third national survey of sexual attitudes and lifestyles (Natsal-3). BMC Public Health. (2016) 16:525. 10.1186/s12889-016-3149-z 27386950 PMC4936006

[47] EMIS Network EMIS 2010: The European Men-Who-Have-Sex-with-Men Internet Survey. FINDINGS from 38 Countries. European Centre for Disease Prevention and Control (2013). Retrieved from https://ecdc.europa.eu/sites/portal/files/media/en/publications/Publications/EMIS-2010-european-men-who-have-sex-with-men-survey.pdf (accessed July 19, 2020).

[48] Bremer V Dudareva-Vizule S Buder S der Heiden M Jansen K. Sexuell übertragbare Infektionen in Deutschland. Bundesgesundheitsblatt-Gesundheitsforschung-Gesundheitsschutz. (2017) 60:948–57. 10.1007/s00103-017-2590-1 28741188

[49] Poethko-Müller C Zimmermann R Hamouda O Faber M Stark K Ross RS. Epidemiology of hepatitis A, B, and C among adults in Germany: Results of the German health interview and examination survey for Adults (DEGS1). Bundesgesundheitsblatt Gesundheitsforschung Gesundheitsschutz. (2013) 56:707–15. 10.1007/s00103-013-1673-x 23703489

[50] Burgess K Parkhill G Wiggins J Ruth S Stoovè M. Re-wired: treatment and peer support for men who have sex with men who use methamphetamine. Sexual Health. (2018) 15:157–9. 10.1071/SH17148 29754597

[51] Schecke H Lea T Bohn A Kohler T Sander D Scherbaum N. Crystal methamphetamine use in sexual settings among German men who have sex with men. Front Psychiatry. (2019) 10:886. 10.3389/fpsyt.2019.00886 31866883 PMC6910085

[52] Bakker I Knoops L. Towards a continuum of care concerning chemsex issues. Sexual Health. (2018) 15:173–5. 10.1071/SH17139 29402377

